# Ginseng Berry Extract Attenuates Dextran Sodium Sulfate-Induced Acute and Chronic Colitis

**DOI:** 10.3390/nu8040199

**Published:** 2016-04-05

**Authors:** Wei Zhang, Li Xu, Si-Young Cho, Kyung-Jin Min, Tatsuya Oda, LiJun Zhang, Qing Yu, Jun-O Jin

**Affiliations:** 1 Shanghai Public Health Clinical Center, Shanghai Medical College, Fudan University, Shanghai 201508, China; weiwei061215@126.com (W.Z.); wendengxvli@126.com (L.X.); zhanglijun1221@163.com (L.Z.); 2 R & D Unit, AmorePacific Corporation, 1920 Yonggudae-ro, Giheung-gu, Yongin-si, Gyeonggi-do 17074, Korea; csy1010@amorepacific.com; 3 Department of Biological Sciences, Inha University, Incheon 22212, Korea; minkj@inha.ac.kr; 4 Division of Biochemistry, Faculty of Fisheries, Nagasaki University, Nagasaki 55001, Japan; t-oda@nagasaki-u.ac.jp; 5 Department of Immunology and Infectious Diseases, The Forsyth Institute, 245 First Street, Cambridge, MA 02142, USA; Qyu@forsyth.org; 6 Department of Oral Medicine, Infection and Immunity, Harvard School of Dental Medicine, Boston, MA 02115, USA

**Keywords:** ginseng berry extract, mouse colitis, intestinal dendritic cell, intestinal macrophage

## Abstract

This study investigates the *in vivo* functions of ginseng berry extract (GB) as a therapy for dextran sodium sulfate (DSS)-induced colitis. C57BL/6 mice were given drinking water containing DSS (3%) for eight days to induce acute colitis. At the same time, the mice received an oral dose of GB (50 mg/kg) once daily. The GB-treated mice were less susceptible to the development of acute colitis than were control mice treated with saline, as determined by weight loss, disease activity, and colon histology. The administration of GB to DSS-treated mice also reduced the numbers and inhibited the activation of colon-infiltrating T cells, neutrophils, intestinal CD103^−^CD11c^+^ dendritic cells (cDCs), and macrophages. In addition, GB treatment promoted the migration of CD103^+^CD11c^+^ cDCs and expansion of Foxp3^+^ regulatory T cells in the colons of DSS-treated mice. Similarly, in the DSS-induced chronic colitis model, GB treatment improved the macroscopic and histological appearance of the colon wall when compared to untreated control mice, as indicated by longer colon length and lower histological scores. This is the first report to show that oral administration of GB suppresses immune activation and protects against experimentally induced colitis.

## 1. Introduction

Ginseng root has been used in Asian countries as a traditional medicine for various diseases, such as cancer, autoimmune disorders, and atherosclerosis [1]. Previous reports have shown that ginseng root extract (GR) has strong anti-inflammatory properties [2,3] and can attenuate dextran sodium sulfate (DSS)-induced colitis and colon cancer in mice [4]. Ginseng berry extract (GB) shows similar anti-inflammatory effects and can ameliorate streptozotocin-induced diabetes in mice [5]. The ginsenosides in GR are the active components and contribute greatly to its pharmacological activities [6]. Previous studies have shown that the ginsenoside profile of GB differs from that of GR, and that the ginsenoside content is four to six times higher in GB than in GR [6,7]. Moreover, GB shows a greater potency than GR in inducing anti-hyperglycemic activities [8] and relaxation effects on the penile corpus cavernosum smooth muscle [7]. However, the *in vivo* anti-inflammatory effects of GB, and especially on DSS-induced colitis, have not been investigated or compared to the effects of GR.

The etiology of inflammatory bowel diseases (IBDs), such as ulcerative colitis (UC) and Crohn’s disease (CD), still remains unknown. Interactions between immunologic, genetic, and environmental factors and the intestinal microflora may contribute to the pathogenesis of IBDs [9]. Dysregulated immune responses in the intestinal mucosa cause an overproduction of pro-inflammatory cytokines, such as IL-6, IL-17, IFN-γ, and TNF-α, and contribute to the development of colitis [10,11]. Immune cells, including dendritic cells (DCs), macrophages, neutrophils, and T cells, together with the pro-inflammatory cytokines, collaborate to perpetuate and sustain the inflammatory responses in the colon, eventually leading to tissue damage and the development of colitis [12].

Inhibition of myeloid cell migration in IL-10-knockout mice and depletion of phagocytes ameliorate the development of colitis, suggesting that DCs and macrophages are directly involved in the development of this disease [13]. The DCs isolated from the colon tissue of patients with UC show higher CD40 expression than is observed in DCs from healthy individuals [14]. Moreover, depletion of DCs before the induction of colitis by DSS results in exacerbation of the disease [15,16]. In addition, intestinal CD103^+^ DC subsets are able to induce expansion of Foxp3-expressing regulatory T (Treg) cells [9,15], which prevent inflammation in the colon [17]. By contrast, CD103^−^ DCs promote the generation of T helper (Th) 17 cells [15,18], which enhances the pathogenesis of UC [19]. Thus, the protective/pathogenic role of distinct DC subsets in the intestine remains an active area of investigation.

In this study, we investigated the effect of GB on DSS-induced acute and chronic colitis models and tested the hypothesis that GB has anti-inflammatory and immune-suppressing effects that protect against these diseases.

## 2. Materials and Methods

### 2.1. Animals

C57BL/6 mice were purchased from Shanghai Public Health Clinical Center and kept under pathogen-free conditions. The mice were maintained in a room with controlled temperature (20–22 °C), humidity (50%–60%), and light (12 h:12 h) and were given free access to standard rodent chow and water. This study was carried out in strict accordance with the recommendations in the Guide for the Care and Use of Laboratory Animals of the Shanghai Public Health Clinical Center. The protocol was approved by the Committee on the Ethics of Animal Experiments of Fudan University (Permit Number: SYXK-2010-0098). Mice were sacrificed by CO_2_ inhalation euthanasia, and all efforts were made to minimize suffering.

### 2.2. Chemicals

DSS (molecular weight 36,000–50,000) was purchased from MP Biomedicals (Guang Zhou, China). Freshly harvested four-year-old Korean ginseng (*Panax ginseng* CA Meyer) roots and berries, cultivated in the Chungbuk Province of Korea, were purchased in July 2014, and the species identity was confirmed by Dr. Ki Ho Kim from Biolandkorea Co. (Chungnam, Korea). Voucher specimens (GBP1207 for ginseng berry) were deposited at the herbarium of the College of Environmental and Bioresource Sciences, Chonbuk National University, Korea. Ginseng roots (100 g) were extracted with 600 mL 55% ethanol at 65–68 °C for 24 h, with stirring. This extraction procedure was repeated six times. The pooled filtrates were vacuum evaporated at 50–60 °C to yield 50 g of solid content, which was further purified with 500 mL 88% ethanol at 5–10 °C overnight and centrifuged at 5000× *g* for 10 min. The supernatants were evaporated and spray dried to yield 15 g of ginseng extract [20]. The seeds of the ginseng berries were separated and removed, and the remnants were dried in a hot air stream and then refluxed with 70% ethanol for 10 h. The extract was filtered, concentrated under reduced pressure at 45 °C, and lyophilized to produce a GB powder, which was stored at −20 °C until use. The endotoxin levels in GR and GB were evaluated using a *Limulus* amebocyte lysate (LAL) assay kit (Lonza). The GR and GB used in all experiments contained less than 0.1 endotoxin unit/mL.

### 2.3. Comparison and Standardization of Ginsenoside Composition

Minerals were determined by digesting the ash from ginseng roots and berries with 3M hydrochloric acid, followed by atomic absorption spectrophotometry analysis for calcium (Ca), magnesium (Mg), zinc (Zn), and iron (Fe) and the flame photometry analysis for potassium (K) [21]. Soluble and insoluble vitamins were determined by HPLC. Vitamins (Vits.) B1, B2, B6, folic acid, pantothenic acid, and niacin were separated on a SUPELCO Discovery C18 column (150 × 4.6 mm, ID 5 μm) at 35 °C and detected by PDA at 220 nm using a mobile phase of K_2_HPO_4_/methyl alcohol (99/1) at a 1 mL/min flow rate and column pressure of 92 kgf [22]. The insoluble Vits, such as Vit. A, E and K, were separated using n-hexane/isopropanol (99:1) as a mobile phase at a flow rate of 1 mL/min on a Lichrosorb Si60 (250 × 4 mm, ID 5 μm) column at 35 °C [23].

### 2.4. DSS-Induced Acute Colitis

C57BL/6 mice were given 3% DSS in their drinking water for eight consecutive days. The drinking water was changed daily, according to the water volume the mice had consumed during the previous day. Mice were orally administered 50 mg/kg/100 μL of GB, GR, or 0.9% saline (control) during the DSS treatment. The mice in different treatment groups were gender- and age-matched. The mice were sacrificed on day 8 and colonic tissues were harvested for analysis. The disease activity index (DAI) was calculated, as previously described [24]. In brief, the DAI was scored on a scale from 0 to 4 using the following parameters: loss of body weight (0, normal; 1, 0%–5%; 2, 5%–10%; 3, 10%–20%; 4, >20%), stool consistency (0, normal; 2, loose stools; 4, watery diarrhea), and the occurrence of gross blood in the stool (0, negative; 4, positive).

### 2.5. DSS-Induced Chronic Colitis

Chronic colitis was induced by three-cycle administrations of DSS-containing drinking water, with a modification to a previously described method [25]. C57BL/6 mice received 2.5% DSS drinking water for six days, followed by two days of regular drinking water. These mice continued to receive 2.5% DSS drinking water during days 8–14 and 16–22. Mice with chronic DSS colitis were orally treated with GB (50 mg/kg) or 0.9% saline daily on days 1–6, 8–14, and 16–22. The mice in different treatment groups were gender- and age-matched. The mice were sacrificed on day 22 and colon tissue was harvested for further experiments.

### 2.6. Hematoxylin and Eosin Staining

As described in detail previously [26], colon samples were fixed in 4% paraformaldehyde, embedded in paraffin, and sectioned at 5 μm thickness. Sections were then stained with hematoxylin and eosin (H & E) and examined for tissue damage. Colon sections were evaluated using pathology scores for evidence of inflammatory damage, as described previously [27].

### 2.7. Antibodies

The following fluorescence-conjugated antibodies (Abs) were used: CD4 (GK1.5), CD8α (536-7), CD11c (N418), CD86 (GL-1), CD103 (2E7), F4/80 (BM8), MHC class II (M5/114.15.2), Ly-6G (1A8), NK1.1 (PK136), TCR-β (H57-597), TCR-γδ (GL3), anti-IFN-γ (XMG1.2), and anti-IL-17 (TCC11-18H10.1). All antibodies were obtained from BioLegend (San Diego, CA, USA).

### 2.8. Preparation of Lamina Propria (LP) Single Cell Suspensions

The LP single cell suspensions were prepared as described in detail previously [27]. In brief, colons were isolated and washed twice in ice-cold PBS. The tissues were cut open longitudinally, and mucus and gross debris were quickly removed by covering the specimen with dry paper towels. The samples were cut into 0.5–1 cm pieces. Intestinal epithelial cells (IECs) were separated from intestinal pieces by incubating in 0.15% DTT-HBSS (both from Sigma-Aldrich) buffer, with shaking, for 30 min at 37 °C. The released IECs were removed by filtration through a mesh screen. After epithelial cell removal, the LP cells were collected by mincing the remaining tissue into 1 to 2 mm pieces, followed by digestion with 2% FBS containing collagenase (Sigma-Aldrich), with shaking, for 30 min at 37 °C. The cells were filtered through a 100 μm nylon mesh, washed, and the pellet was re-suspended in RPMI-1640 and layered over 1.077 Histopaque (Sigma-Aldrich). After centrifugation at 1700× *g* for 10 min, the light density fraction (<1.077 g/cm^3^) was collected. The single cells were resuspended in culture medium.

### 2.9. Ex Vivo Stimulation of LP Cells and Intracellular Cytokine Staining

As described in detail previously [26], single cell suspensions from the LP were stimulated with phorbol 12-myristate 13-acetate (PMA) (50 ng/mL) and ionomycin (1 μM; both from Calbiochem) for 4 h, with the addition of monensin (eBioscience, San Diego, CA, USA) in the final 2 h. Cells were then stained for NK1.1, Ly-6G, TCR-β, and TCR-γδ to determine the phenotypes of the infiltrating leukocytes. For intracellular cytokine staining, LP cells were surface stained with CD4, CD8, and TCR-β, and then fixed and permeabilized with Cytofix/Cytoperm buffer (eBioscience, San Diego, CA, USA), followed by incubation with anti-IL-17 and anti-IFN-γ antibodies in Perm/Wash buffer (eBioscience, San Diego, CA, USA) for 30 min. Control staining with isotype control IgGs was performed in all experiments.

### 2.10. Intestinal Macrophage and DC Analysis

As described in detail previously [27], single cell suspensions from the colon were incubated for 30 min with the following fluorescence-conjugated monoclonal antibodies (mAbs): anti-CD45, anti-CD11c, anti-MHC class II, anti-CD103, and anti-F4/80. The CD11c^+^CD103^+^F4/80^−^ and CD11c^+^CD103^−^F4/80^−^ cells in CD45^+^MHC class II^+^ cells were defined as migratory cDCs and resident cDCs, respectively [27,28,29]. The CD11c^+^F4/80^+^ cells were defined as macrophages. Analysis was carried out on a FACS Aria II instrument (Becton Dickinson, Franklin Lakes, NJ, USA).

### 2.11. Real-Time qPCR

Total RNA was reverse transcribed into cDNA using Oligo (dT) and M-MLV reverse transcriptase (Promega). The cDNA was subjected to real-time PCR amplification (Qiagen) for 40 cycles with annealing and extension temperature at 60 °C, on a LightCycler 480 Real-Time PCR System (Roche). Primer sequences were: β-actin forward, 5′-TGGATGACGATATCGCTGCG-3′; reverse, 5′-AGGGTCAGGATACCTCTCTT-3′, IFN-γ forward, 5′-GGATGCATTCATGAGTATTGC-3′; reverse, 5′-CTTTTCCGCTTCCTGAGG-3′, T-bet forward, 5′-CAACAACCCCTTTGCCAAAG-3′; reverse, 5′-TCCCCCAAGCATTGACAGT-3′, IL-17A forward, 5′-GCGCAAAAGTGAGCTCCAGA-3′; reverse 5′-ACAGAGGGATATCTATCAGGG-3′, RORγt forward, 5′-CCGCTGAGAGGGCTTCAC-3′; reverse 5′-TGCAGGAGTAGGCCACATTACA-3′, IL-6 forward, 5′-ACGATGATGCACTTGCAGA-3′; reverse, 5′-GAGCATTGGAAATTGGGGTA-3′, IL-12p40 forward, 5′-CACATCTGCTGCTCCACAAG-3′; reverse 5′-CCGTCCGGAGTAATTTGGTG-3′, IL-23p19 forward, 5′-CTCTCGGAATCTCTGCATGC-3′; reverse 5′-ACCATCTTCACACTGGATACG-3′.

### 2.12. ELISA

Mouse IL-6, IL-12p70, IL-23, IFN-γ, and IL-17 (Biolegend, San Diego, CA, USA) concentrations in whole colonic homogenates or T cell cultured medium were determined using ELISA kits according to the manufacturer’s protocols.

### 2.13. In Vitro T Cell Stimulation

As described in detail previously [30], naïve-enriched CD4 T cells were purified from spleen and mesenteric lymph nodes by negative selection using mouse CD4^+^ T cell isolation kits (Miltenyi) and placed at 1 × 10^6^ cells per well in 24-well plates that were coated with anti-CD3 (1 μg/mL) and anti-CD28 (1 μg/mL). Cytokines were added to the cultures as indicated. IL-1β, IL-6, IL-12, and IL-23 were used at 20, 20, 50, and 10 ng/mL, respectively and GB was added at 100 μg/mL. After three days of culture, cells were harvested for analysis of effector T cell differentiation.

### 2.14. Statistical Analysis

Results are expressed as the mean ± standard error of the mean (SEM). The statistical significance of differences between experimental groups was calculated using analysis of variance with a Bonferroni post-test or an unpaired Student’s *t*-test. All *p*-values < 0.05 were considered significant.

## 3. Results

### 3.1. The Micronutrient Content is Higher in GB Than in GR

Previous studies have shown that the ginsenoside content is higher in GB than GR, especially for ginsenosides Re and Rg1 [7]. However, the micronutrient composition in GB and GR has not been compared previously. Our analysis of the micronutrients in GB and GR, including Ca, Mg, Zn, Fe, K, Vit., pantothenic acid, niacin and folate, revealed a fourfold higher total amount of micronutrients in GB than in GR. The amounts of Fe, Zn, Vit., niacin, and pantothenic acid were also five- to ten-fold higher in GB than in GR. Some Vits., notably Vit. A, Vit. B6, Vit. E, Vit. B2, and folate, were detected only in GB but not in GR (Table 1). Overall, the types and amounts of micronutrients were much higher in GB than in GR.

### 3.2. GB Attenuates DSS-Induced Acute and Chronic Colitis

**Figure 1:**
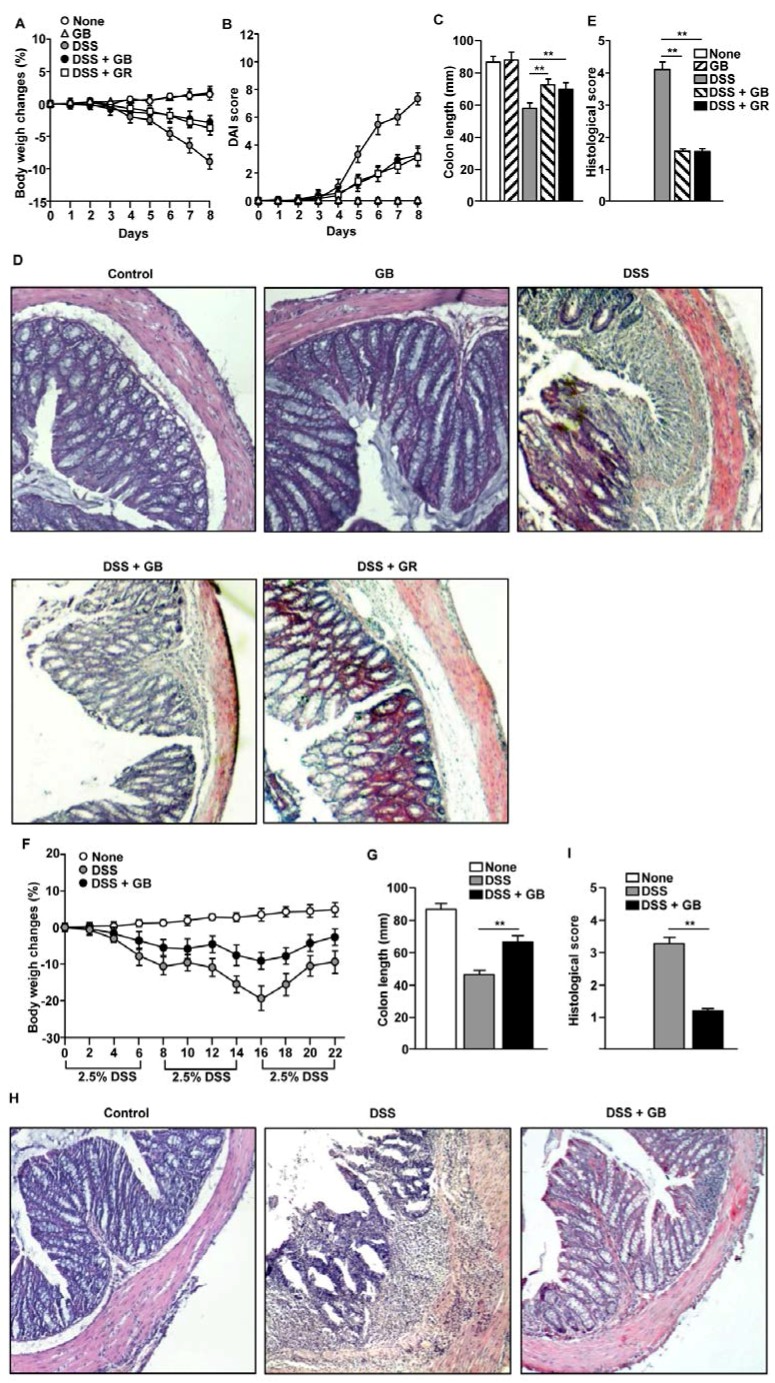
Ginseng berry extract (GB) treatment attenuates DSS-induced colitis. (**A** to **E**) Dextran sodium sulfate (DSS; 3%) was added to the drinking water of C57BL/6 mice and the mice were orally treated with GB, ginseng root extract (GR) or 0.9% saline for eight days. (**A**) Body weight changes in control (no treatment), GB, DSS, DSS + GB, and DSS + GR groups (*n* = 9); (**B**) The disease activity index (DAI) was scored using stool consistency, the presence or absence of fecal blood, and weigh loss; (**C**) Colon length of colitis mice at day 8; (**D**) Hematoxylin and eosin (H & E) staining images are shown at 80× magnification; (**E**) Histological scores were evaluated by immune cell infiltration and colon damage. Data are the average or representative of analyses of three independent experiments (total *n* = 9/group); (**F** to **H**) C57BL/6 mice were given drinking water containing 2.5% DSS for three cycles to induce chronic colitis. Some of the DSS-treated mice also received daily oral administration of GB. Non-treated control mice drank only distilled water. Mice were sacrificed at day 22 and analyzed for disease parameters; (**F**) Body weight changes (*n* = 9); (**G**) Colon length; (**H**) Colon sections stained with H & E from three groups of mice at the same magnification (80×); (**I**) Histological scores. Data are representative of/or the average of analyses of three independent experiments (total *n* = 9). Data shown are the mean ± SEM. ** *p* < 0.01.

Since GR has a known inhibitory effect on DSS-induced colitis [4], and since GB had higher levels of nutrients than GR, we examined whether GB could also ameliorate DSS-induced acute colitis. The mice treated with DSS alone exhibited substantial weight loss of almost 10% of their body weight from day 4 to day 8. The body weight loss was lower in the DSS-treated mice that received concomitant GB administration than in mice treated with DSS alone. The effect of GR was similar to that of GB (Figure 1A). The DAI scores showed markedly lower disease severity in the GB- and GR-treated mice than in those treated with DSS alone (Figure 1B). In addition, the shortening of colon length induced by DSS administration was markedly inhibited by GB and GR treatment (Figure 1C). The colon tissues of mice treated with DSS showed substantial infiltration of inflammatory cells, loss of crypts, and reduction of goblet cells, whereas these changes were markedly inhibited by GB and GR treatment (Figure 1D,E). Mice treated with GB but without DSS administration showed similar tissue parameters to those seen in non-treated control mice. The GB and GR treatments had similar protective effects on acute colitis, as both reduced the body weight loss and colon inflammation induced by DSS.

We determined whether GB attenuates DSS-induced chronic colitis by feeding C57BL/6 mice with drinking water containing 2.5% DSS for six days, followed by two days of regular drinking water for three cycles. In this chronic colitis model, the body weight in mice treated with DSS gradually decreased until day 16 and was partially recovered on day 22. In comparison, DSS-treated mice that also received oral GB showed markedly less body weight loss (Figure 1F) and greater colon length (Figure 1G). In addition, DSS-induced colon tissue damage and immune cell infiltration were substantially decreased by GB treatment (Figure 1H,I).Thus, oral administration of GB appeared to result in effective attenuation of DSS-induced acute and chronic colitis.

### 3.3. GB Reduces the Number of T Cells and Neutrophils in the Colon

**Figure 2:**
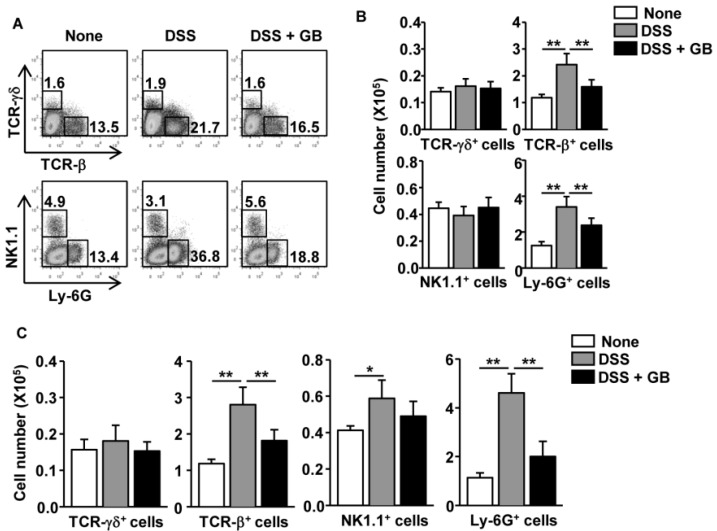
Ginseng berry extract (GB) reduces the number of T cells and neutrophils in the colons of mouse colitis models. Dextran sodium sulfate (DSS; 3%) was added to the drinking water of C57BL/6 mice to induce colitis and the mice were orally treated with GB or 0.9% saline for eight days. Colon tissue was harvested and used to prepare single cell suspensions. (**A**) The frequency of colon-infiltrating TCR-β^+^, TCR-γδ^+^, NK1.1^+^ and Ly-6G^+^ cells cells is indicated by the number shown in each plot; (**B**) Mean of absolute numbers in colon-infiltrating TCR-β ^+^, TCR-γδ^+^, NK1.1^+^, and Ly-6G^+^ cells; (**C**) Chronic colitis was induced in C57BL/6 mice as described in Figure 2F. Mean of absolute numbers of colon-infiltrating TCR-γδ^+^, TCR-β^+^, NK1.1^+^, and Ly-6G^+^ cells. Data are representative of/or the average of analyses of three independent experiments (total *n* = 9). Data shown are the mean ± SEM. * *p* < 0.05, ** *p* < 0.01.

We then examined whether GB reduces the increased in numbers of colon-infiltrating immune cells occurring in response to DSS administration. We harvested colons on day 8 of DSS administration and analyzed the infiltrating cells by flow cytometry. DSS administration caused a substantial increase in the frequency of T cell receptor (TCR)-β^+^ cells and Ly-6G^+^ neutrophils (Figure 2A, middle panel). GB treatment significantly reduced the percentages of TCR-β^+^ cells and Ly-6G^+^ neutrophils in DSS-treated mice (Figure 2A, right panel). The absolute numbers of colon-infiltrating TCR-β^+^ cells and Ly-6G^+^ neutrophils were also significantly decreased by GB treatment (Figure 2B).

The subsequent measurement of colon-infiltrating immune cells in the chronic colitis model revealed similar results to those found in the acute colitis model: the number of TCR-β^+^ T cells and Ly-6G^+^ neutrophils in the colon of DSS-treated mice were significantly decreased by GB treatment, whereas the numbers of NK1.1^+^ cells were not significantly affected (Figure 2C). Thus, GB treatment during DSS induction of colitis appeared to reduce the numbers of colon-infiltrating TCR-β^+^ T cells and neutrophils.

### 3.4. GB Inhibits DSS-Induced Th1 and Th17 Responses

**Figure 3:**
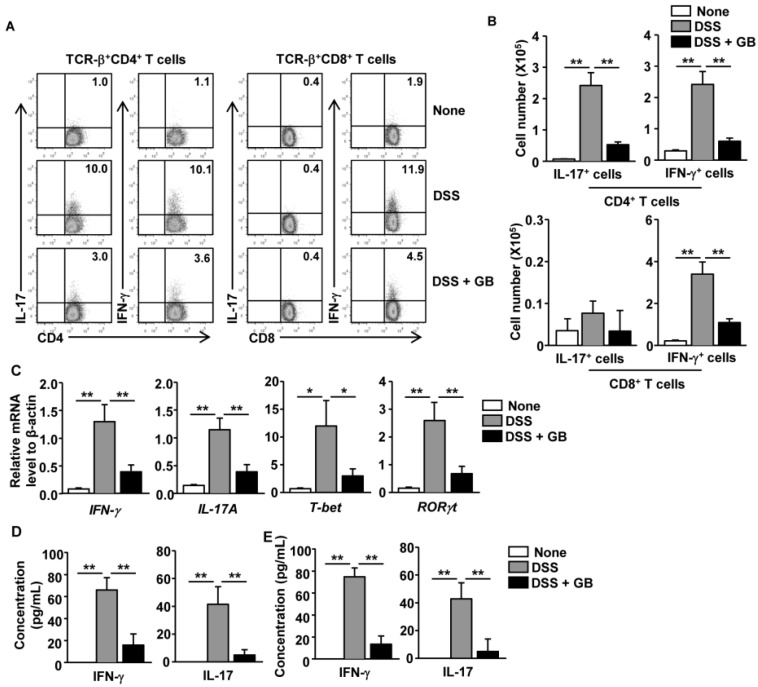
Ginseng berry extract (GB) suppresses Th1 and Th17 responses in dextran sodium sulfate (DSS)-treated mice. DSS (3%) was added to the drinking water of C57BL/6 mice and the mice were orally treated with GB or 0.9% saline for eight days. (**A**) Percentage of IFN-γ^+^ and IL-17^+^ in CD4^+^ (left panels) and CD8^+^ (right panels) T cells, identified by gating on the TCR-β^+^CD4^+^ and TCR-β^+^CD8^+^ cells respectively, within colon-infiltrating mononuclear cells; (**B**) Means of absolute numbers of colon-infiltrating IFN-γ^+^ and IL-17^+^ in CD4^+^ (upper panels) and CD8^+^ (lower panels) T cells are shown; (**C**) Real-time PCR analysis of gene expression in colon tissues; (**D**) Concentrations of IFN-γ and IL-17 in colonic homogenates were determined by ELISA; (**E**) Chronic colitis was induced in C57BL/6 mice, as described in Figure 2F, and the concentration of IFN-γ and IL-17 in colonic homogenates was determined by ELISA. All data are representative of/or the average of analyses of three independent experiments (total *n* = 9). Data shown are the mean ± SEM. * *p* < 0.05, ** *p* < 0.01.

DSS-induced colitis is mediated by Th1 and Th17 responses, resulting in increased numbers of IFN-γ- and IL-17A-producing CD4 T cells in the inflamed colonic tissue [11,31,32]. The Th17 immune response also promotes neutrophil recruitment to the colon during the development of DSS-induced colitis [33,34]. Having demonstrated that the number of TCR-β^+^ cells and Ly-6G^+^ neutrophils in the colon were decreased by GB, we next tested whether GB treatment might inhibit Th1 and Th17 responses in DSS-treated mice. On day 8 of DSS administration, we analyzed intracellular cytokine production in colon infiltrating T cells. The percentages of DSS-induced IFN-γ and IL-17 producing CD4^+^ T cells were markedly decreased by GB treatment (Figure 3A, left panels). The percentages of DSS-induced IFN-γ-producing CD8 T cells, but not IL-17-producing CD8 T cells, were also decreased by GB treatment (Figure 3A, right panels). Accordingly, the absolute numbers of IFN-γ^+^ CD4, IL-17^+^ CD4, and IFN-γ^+^ CD8 T cells in the colon were significantly decreased by GB treatment of DSS-treated mice (Figure 3B). Real-time PCR analysis also showed higher mRNA levels of IFN-γ and IL-17A, and of T-bet and RORγt (the signature transcription factors for Th1 and Th17) in the colons of the DSS-treated mice than in the colons of the untreated control mice (Figure 3C). The DSS-induced increase in these genes was almost completely abolished by GB treatment (Figure 3C). Consistently, DSS-induced production of IFN-γ and IL-17 protein in the colon was also significantly reduced by GB treatment (Figure 3D). The DSS-induced increases in the IFN-γ and IL-17 protein levels in colon homogenates of the chronic colitis model were also almost completely inhibited by GB treatment (Figure 3E). Hence, these data demonstrate that GB inhibited DSS-induced Th1 and Th17 immune responses.

### 3.5. GB Does Not Directly Affect Th1 and Th17 Cell Differentiation

**Figure 4:**
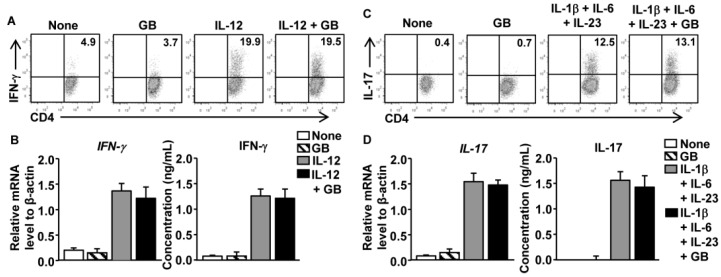
Ginseng berry extract (GB) did not directly inhibit differentiation of Th1 and Th17 cells from naïve CD4 T cells. Purified naïve-enriched CD4 T cells were stimulated with plate-bound anti-CD3 plus anti-CD28 with the indicated cytokines in the presence or absence of GB for 72 h. (**A**) Flow cytometry of IFN-γ expression in CD4 T cells is shown; (**B**) The mRNA levels (left panel) and protein levels of IFN- γ (right panel) are shown; (**C**) Flow cytometry of IL-17 expression in CD4 T cells is shown; (**D**) The mRNA levels (left panel) and protein levels of IL-17 (right panel) are shown. All data are representative of/or the average of analyses of three independent experiments (total *n* = 6).

We directly assessed the effect of GB on CD4 T cells by purifying naïve CD4 T cells and stimulating them *in vitro* with anti-CD3 and anti-CD28 antibodies under Th1- or Th17-skewing conditions, in the presence or absence of GB for three days. Addition of IL-12 to the CD4 T cell cultures promoted the generation of IFN-γ-producing Th1 cells; this response was not affected by GB treatment (Figure 4A). The mRNA and secreted protein levels of IFN-γ showed no considerable decrease in response to GB treatment (Figure 4B). Addition of a combination of IL-1β, IL-6, and IL-23 to CD4 T cell cultures promoted the generation of IL-17-producing Th17 cells; this response was also not altered by GB treatment (Figure 4C). Accordingly, GB treatment did not affect the levels of IL-17 mRNA or secreted protein (Figure 4D). Thus, GB does not appear to have a direct effect on the differentiation of Th1 and Th17 cells from naïve CD4 T cells.

### 3.6. GB Inhibits DSS-Induced Activation of Intestinal DCs and Macrophages and Induces the Expansion of Regulatory T Cells

**Figure 5:**
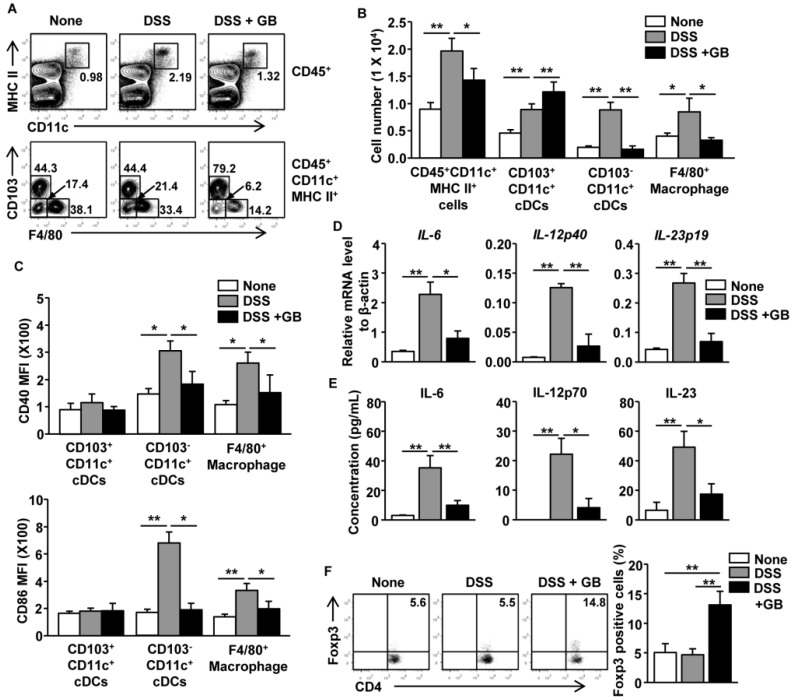
Ginseng berry extract (GB) inhibits recruitment and activation of CD103^-^CD11c^+^ dendritic cells (cDCs) and macrophages in the colon. Dextran sodium sulfate (DSS; 3%) was added to the drinking water of C57BL/6 mice and the mice were orally treated with GB or 0.9% saline for eight days. (**A**) Percentage of CD11c^+^MHC class II^+^ in CD45^+^ cells (upper panels) and CD103^+^, CD103^-^, and F4/80^+^ cells in CD11c^+^MHC class II^+^CD45^+^ cells within colon-infiltrating mononuclear cells as determined by flow cytometry; (**B**) Means of absolute numbers of colon-infiltrating CD45^+^CD11c^+^MHC class II^+^ cells, CD103^+^CD11c^+^ cDCs, CD103^-^CD11c^+^ cDCs, and F4/80^+^ macrophages are shown; (**C**) Mean fluorescence intensity (MFI) of CD40 and CD86 are shown in the indicated cells; (**D**) Expression levels of IL-6, IL-12p40, and IL-23p19 mRNA were measured in colon tissues; (**E**) Concentrations of IL-6, IL-12, and IL-23 in colonic homogenates were determined by ELISA; (**F**) CD4^+^Foxp3^+^ cells are shown in the colon (left panel), mean percentage of Foxp3 positive cells in CD4 T cells (right panel). All data are representative of/or the average of analyses of three independent experiments (total *n* = 6). Data shown are the mean ± SEM. * *p* < 0.05, ** *p* < 0.01.

Intestinal DCs and macrophages are thought to play a central role in the pathogenesis of colitis [10]. Innate immune cells, such as DCs and macrophages, also produce pro-inflammatory cytokines that promote effector T cell generation [18,35]. Our observation that GB treatment does not directly affect differentiation of Th1 and Th17 cells prompted us to examine whether GB could inhibit the activation of intestinal DCs and macrophages during the DSS induction of colitis. DSS induced substantial increases in the frequency and cell numbers of CD11c^+^MHC class II^+^ cells in CD45^+^ cells of the colon, whereas those increases were reduced by GB treatment (Figure 5A upper panel and 5B). The frequency and cell numbers of CD103^−^CD11c^+^ resident cDC subsets and F4/80^+^ macrophages were also elevated by DSS administration, and again these increases were decreased by GB treatment (Figure 5A lower panel and 5B). DSS also induced the upregulation of CD40 and CD86 in CD103^−^CD11c^+^ resident cDCs and F4/80^+^ macrophages, while these responses were diminished by GB treatment (Figure 5C). However, these co-stimulatory molecules were not upregulated in CD103^+^CD11c^+^ migratory cDCs during the DSS treatment (Figure 5C). The DSS-induced increase in IL-6, IL-12p40, and IL-23p19 mRNA and protein, which were mainly generated by DCs and macrophages, were also almost completely inhibited by GB treatment (Figure 5D,E).

The GB treatment of DSS-treated mice led to significant increases in the frequency and cell numbers of CD103^+^CD11c^+^ migratory cDCs (Figure 5A lower panel and 5B). Since these cDCs promote migration of Foxp3^+^ Treg cells in the colon [15,36], we next measured Foxp3^+^ Treg cell infiltration in the mouse colons. We found that GB treatment induced significant increases in the numbers of Foxp3^+^ Treg cells in the colon in DSS-treated mice, when compared to control untreated mice or mice treated with DSS alone (Figure 5F). Thus, GB treatment appeared to suppress the DSS-induced activation of intestinal CD103^−^CD11c^+^ resident cDCs and macrophages and to induce migration or expansion of CD103^+^CD11c^+^ migratory cDCs and Foxp3^+^ Treg cells in the colon.

## 4. Discussion

A previous study showed that GR attenuates DSS-induced colitis and colon cancer in mice [4]. However, the effect of GB on DSS-induced colitis has not been previously investigated, nor have the anti-inflammatory effects of GB and GR been compared. In this study, we demonstrated that GB and GR treatment had a largely similar inhibitory effect on DSS-induced colitis. We also showed that the amounts of micronutrient components are higher in GB than in GR. Ginseng extracts contain medicinal ingredients, including saponin and ginsenosides. The ginsenosides are the major active components in both GB and GR [37], but the two extract types have different ginsenoside profiles, as GB contains higher levels of ginsenosides-Re and Rg1 compared to GR [7,38].

Many factors alter nutrient intake in patients with IBD, and vit. and mineral deficiencies have been well documented in these patients [39], which can result from decreased food intake and increased intestinal losses [40]. Thus, medical nutrition therapy that provides vits. and minerals, including folate, Ca, Zn, Fe, and Mg, has been proposed for as both a primary and supportive treatment for IBD [40]. However, the protective effect of nutrients against IBD is not well characterized. Possible explanations for the protective effect are that nutrients promote stability of the composition of the intestinal microbiota [41] or that they directly inhibit immune activation [42]. In this study, we found much higher amounts of micronutrients (*i.e.*, vits, folate, Ca, Zn, Fe, and Mg) in GB than in GR, and yet both extracts showed similar inhibitory effects on DSS-colitis. In addition, Vit. D is required for protection against colitis in the mouse model [43], whereas Vit. B deficiency is protective against DSS-colitis [44]. Although Vit. B levels were much higher in GB than GR, GB still efficiently protected the mice from colitis and its effects were similar to those of GR. Therefore, the high amounts of Vit. B contained in the GB may suppress the protective effect against DSS-colitis, despite the higher levels of other micronutrients found in GB compared to GR. At present, no evidence indicates that the provision of high amounts of micronutrients could significantly increase the micronutrients existing in the diet. Therefore, further studies are required to determine the effects of micronutrients in these extracts. The alterations in microbiota composition and elevations of micronutrients in the diet in response to these extracts will be the focus of future studies.

UC is a chronic relapsing and remitting inflammatory condition of the intestine, with high prevalence in developed countries. Interactions between genetic and environmental factors, together with a dysregulated immune response, may be the cause of UC pathogenesis [9]. This dysregulation of the immune response is thought to be due to the imbalance of activated immune cells, such as DCs, macrophages, NK cells, neutrophils, and T cells. The Th1 and Th17 responses are apparently the main contributors to the pathogenesis of UC [45,46]. The results from the current study indicate that GB does not directly inhibit effector T cell differentiation and that T cells are not the main target cells of GB. Therefore, we speculate that the *in vivo* inhibition of T cell differentiation by GB treatment results from a decreased activation and tissue migration of intestinal DCs and macrophages, which are the main target cells of GB. GB also prevents the recruitment of neutrophils to the colon during DSS-induction of colitis. Since the migration of neutrophils into the colon tissue contributes to the exacerbation of UC disease activities [47], and since Th17 cells can recruit and stimulate neutrophils via activation of local tissues [33,34], our data are in line with previous studies showing that suppression of Th17 responses by GB may inhibit neutrophil activation and reduce migration into the colon.

DSS induces apoptosis of the colonic epithelium in the mouse model, which promotes inflammation in the colon [48]. Our examination of GB effects on DSS-induced apoptosis of colonic epithelium revealed no inhibition of DSS-induced apoptosis of colonic epithelium by GB (Figure S1). We found that GB treatment suppresses the development of colitis and inhibits immune activation, but whether immune suppression is the key mechanism by which GB inhibits colitis remains unclear. GB could possibly cause a direct suppression of non-immunological events that are critical for the pathogenesis of DSS-induced colitis, such as the perturbation of the intestinal epithelial tissue integrity/barrier function, thereby protecting against this disease. Therefore, more studies are required to distinguish among these possibilities and to define the precise cellular and molecular mechanisms underlying the anti-inflammatory effect of GB in DSS-colitis.

Antigen presenting cell (APC) populations in the intestine contribute to maintaining the balance between pathogenic and tolerogenic immune responses. Different subsets of intestinal macrophages and DCs can have either pathogenic or protective roles in mouse models of colitis [10,13]. We found that GB treatment decreased the recruitment of intestinal CD103^-^CD11c^+^ cDCs and macrophages in DSS-induced colitis. Similar to results from human UC studies [14], cDCs and macrophages in DSS-treated mice also expressed higher levels of CD40 and CD86 than did those from control mice, and this effect was almost completely eliminated by GB treatment. Since, M1-polarized pro-inflammatory macrophages contribute to tissue inflammation and UC pathogenesis [49,50], the inhibition of M1 macrophage activation may contribute to the amelioration of colitis by GB. Interestingly, in contrast to CD103^-^CD11c^+^ cDCs, the CD103^+^CD11c^+^ cDCs did not show upregulated expression of CD40 and CD86 during DSS-induction of acute colitis, although the numbers of these cells in the colon were increased. The CD103^+^CD11c^+^ cDCs in the intestine have the unique ability to induce gut homing phenotypic changes [15,28,51]. These cells functionally produce high levels of retinoic acid, which can promote the generation of Treg cells [52]. While CD103^−^CD11c^+^ cDCs have the capacity to prime CD4 and CD8 T cell responses, they do not have the ability promote the generation of gut homing T cells [15,51]. Therefore, the GB-induced increase in CD103^+^CD11c^+^ cDCs in the intestine may directly promote gut homing T cell responses, such as Treg cell expansion and migration, which may directly protect against DSS-induced inflammation of the colon.

Although our previous study found that intraperitoneal (*i.p.*) injection of GB promotes spleen DC activation [53], ginseng products are commonly taken orally. During the course of this study, we also found that, in contrast to oral administration of GB, *i.p.* injection of GB was unable to reduce the disease severity of DSS-induced colitis. In line with our observation, others have also reported that oral administration of fucoidan ameliorates colitis [54], but intravenous (*i.v.*) injection of fucoidan induces systemic immune activation [55,56]. Therefore, the *in vivo* bioactivities of GB vary depending on the route of its administration and its subsequent tissue distribution. Our future studies will investigate how different injection routes of GB cause different outcomes in the immune responses and determine the cellular and molecular basis underlying these differences.

DSS-induced colitis has been a widely-used and accepted animal model for studying human UC [57,58]. However, this mouse model does exhibit different immune responses compared to human UC, as adaptive immune responses do not play a prominent role in the development of UC disease [57,59]. Hence, although GB has a clear colitis-inhibiting effect in the mouse model, whether it exerts similar effects on human UC still requires thorough investigation. In addition, an interesting aspect for future studies is assessment of whether GB can be used together with other therapies to enhance the efficacy of existing treatments.

## 5. Conclusions

In conclusion, the present study demonstrates a crucial protective function of oral administration of GB in DSS-induced acute and chronic colitis in mice, which is accompanied by reduced numbers and inhibited activation of DCs, macrophages, T cells, and neutrophils in the colon. Further investigations are warranted to determine the effect of GB on human colitis, which may provide critical knowledge for the development of new therapeutic strategies for this disease.

## Figures and Tables

**Table 1:** Composition of ginsenoside in ginseng berry extract (GB) and ginseng root extract (GR).

	K	Ca	Fe	Mg	Zn	Folate	PA	Niacin	Vit.				
									A	B1	B2	B6	E
GB	5860	820	59.3	354	178	345	5.8	5.8	213	12.3	8.4	10.5	23.6
GR	1460	340	9.8	160	2	n.d.	0.7	1	n.d.	0.2	n.d.	n.d.	n.d.

The micronutrient component of GB and GR per 100 g was analyzed as shown in the Materials and Methods. Numbers in the figure show the concentration of the indicated micronutrient (mg/100 g).

## Supplementary Files

Supplementary File 1

## Abbreviations

The following abbreviations are used in this manuscript:

GB: ginseng berry extract
DSS: dextran sodium sulfate
UC: ulcerative colitis
GR: ginseng root extract
IBD: inflammatory bowel diseases
CD: Crohn’s disease
DC: dendritic cell
Th: T helper
Treg: regulatory T
LAL: limulus amebocyte lysate
Ca: calcium
Mg: magnesium
Zn: zinc
Fe: iron
K: potassium
Vit.: vitamin
DAI: disease activity index
PMA: phorbol 12-myristate 13-acetate
