# Supplementary material for: Ginseng Berry Extract Attenuates Dextran Sodium Sulfate-Induced Acute and Chronic Colitis
Source: Nutrients. 2016 Apr 5;8(4):199. doi: 10.3390/nu8040199 (PMC4848668; doi:10.3390/nu8040199)
Supplement: Supplementary File 1 [file nutrients-08-00199-s001.docx]

Ginseng Berry Extract Attenuates Dextran Sodium Sulfate-Induced Acute and Chronic Colitis

Wei Zhang, Li Xu, Si-Young Cho, Kyung-Jin Min, Tatsuya Oda, LiJun Zhang, Qing Yu and Jun-O Jin


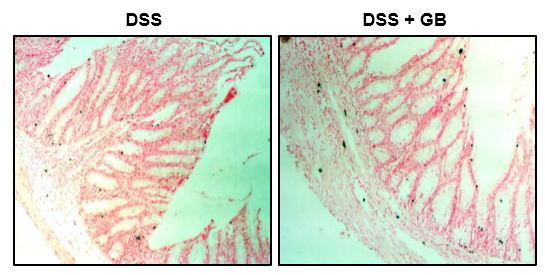


**Figure S1.** GB did not inhibit DSS-induced apoptosis in colon epitherium. *In situ* detection of cell apoptosis in colon from mice treated with DSS or DSS and GB for 3 days. Data are representative of 5 individual mice for each treatment group (2–3 mice per experiment, total 2 independent experiments).
